# Computed tomography in patients with sepsis presenting to the emergency department: exploring its role in light of patient outcomes

**DOI:** 10.1007/s00330-024-10701-y

**Published:** 2024-04-09

**Authors:** Julian Pohlan, Martin Möckel, Anna Slagman, Hannah Tenenbaum, Jules Stolz, Kerstin Rubarth, Johannes Winning, Michael Bauer, Konrad Reinhart, Angelika Stacke, Marc Dewey, Myrto Bolanaki

**Affiliations:** 1 Department of Radiology, Charité – Universitätsmedizin Berlin, corporate member of Freie Universität Berlin and Humboldt Universität Zu Berlin, Charitéplatz 1, 10117 Berlin, Germany; 2 Berlin Institute of Health at Charité – Universitätsmedizin Berlin, Charitéplatz 1, 10117 Berlin, Germany; 3 Department of Emergency and Acute Medicine, Charité – Universitätsmedizin Berlin, corporate member of Freie Universität Berlin and Humboldt Universität Zu Berlin, Augustenburger Platz 1, 13353 Berlin, Germany; 4 Institute for Biometry and Clinical Epidemiology, Charité – Universitätsmedizin Berlin, corporate member of Freie Universität Berlin and Humboldt Universität Zu Berlin, Charitéplatz 1, 10117 Berlin, Germany; 5 Department of Anaesthesiology and Intensive Care Medicine, Jena University Hospital, Am Klinikum 1, 07747 Jena, Germany; 6 Department of Anesthesiology and Operative Intensive Care Medicine, Charité – Universitätsmedizin Berlin, corporate member of Freie Universität Berlin and Humboldt Universität Zu Berlin, Augustenburger Platz 1, 13353 Berlin, Germany; 7 Ernst-Abbe-Hochschule, University of Applied Sciences, Carl-Zeiss-Promenade 2, 07745 Jena, Germany

**Keywords:** CT, Sepsis, Emergency department, Diagnostic accuracy, Mortality

## Abstract

**Objectives:**

This study aimed to explore the role of CT in septic patients presenting to the emergency department (ED).

**Materials and methods:**

We performed a retrospective secondary analysis of 192 septic patients from a prospective observational study, i.e., the “LIFE POC” study. Sepsis was diagnosed in accordance with the Sepsis-3 definition. Clinical and radiological data were collected from the hospital administration and radiological systems. Information on mortality and morbidity was collected. Time-to-CT between CT scan and sepsis diagnosis (ttCTsd) was calculated. Diagnostic accuracy was assessed with the final sepsis source as reference standard. The reference standard was established through the treating team of the patient based on all available clinical, imaging, and microbiological data.

**Results:**

Sixty-two of 192 patients underwent a CT examination for sepsis focus detection. The final septic source was identified by CT in 69.4% (*n* = 43). CT detected septic foci with 81.1% sensitivity (95% CI, 68.0–90.6%) and 55.6% specificity (95% CI, 21.2–86.3%). Patients with short versus long ttCTsd did not differ in terms of mortality (16.1%, *n* = 5 vs 9.7, *n* = 3; *p* = 0.449), length of hospital stay (median 16 d, IQR 9 d 12 h–23 d 18 h vs median 13 d, IQR 10 d 00 h–24 d 00 h; *p* = 0.863), or duration of intensive care (median 3d 12 h, IQR 2 d 6 h–7 d 18 h vs median 5d, IQR 2 d–11 d; *p* = 0.800).

**Conclusions:**

Our findings show a high sensitivity of CT in ED patients with sepsis, confirming its relevance in guiding treatment decisions. The low specificity suggests that a negative CT requires further ancillary diagnostic tests for focus detection. The timing of CT did not affect morbidity or mortality outcomes.

**Clinical relevance statement:**

In patients with sepsis who present to the ED, CT can be used to identify infectious foci on the basis of clinical suspicion, but should not be used as a rule-out test. Scientific evidence for the optimal timing of CT beyond clinical decision-making is currently missing, as potential mortality benefits are clouded by differences in clinical severity at the time of ED presentation.

**Key Points:**

• *In patients with sepsis who present to the ED, CT for focus identification has a high sensitivity and can thereby be valuable for patient management.*

• *As the specificity is considerably lower, a thorough microbiological assessment is important in these cases.*

• *The timing of CT did not affect morbidity and mortality outcomes in this study, which might be due to variability in clinical severity at the time of ED presentation.*

**Supplementary Information:**

The online version contains supplementary material available at 10.1007/s00330-024-10701-y.

## Introduction

Sepsis is a life-threatening condition defined by organ dysfunction due to infection [1]. Biomarkers such as procalcitonin (PCT) in combination with the quick Sequential Organ Failure Assessment (qSOFA) score can play an important role in early identification of sepsis as we reported previously [2], and hence prompt initiation of treatment in the emergency department (ED). Prompt administration of antibiotics is one of the most important interventions in the management of sepsis, and this is a first step in resuscitation that should be initiated in the ED in addition to general measures such as fluid administration, blood sampling for culture, and circulatory support. Early empiric anti-infective therapy improves outcomes for septic patients and should begin in the first hour whenever there is a high likelihood of sepsis [3]. In identifying the appropriate antibiotic agent, various factors need to be taken into consideration by the emergency physician including previous antibiotic treatment, immunosuppression, previous microbiological test results, and most importantly, the possible source of the infection. Thus, the search for the infectious focus should start promptly [4].

However, identification of the septic focus may be challenging for ED physicians. Especially in patients presenting with septic encephalopathy, an infectious focus may be difficult to pinpoint clinically since past medical history may not be available at the time of presentation [5, 6]. Microbiological analysis of blood and other body fluids is of high importance to isolate causative infectious agents, specifically bacteria or fungi [1, 7], but requires time for culture whereas imaging results are available much more quickly [8, 9].

The choice of the imaging method to identify or exclude a potential infectious focus depends not only on the suspected site of infection, but also on the local availability of imaging facilities, the competence and experience of the medical staff, and of course, individual patient characteristics. Chest x-ray or focused ultrasound can be used as first-line imaging options, but their diagnostic accuracy can be limited, e.g., by obesity or in altered mentation cases poor patient compliance [10, 11]. Computed tomography (CT) is commonly used when the infectious focus is unclear in septic patients [12]. CT provides fast three-dimensional imaging data which allows for the localization of an infectious focus [13]. Additionally, contrast-enhanced CT may improve the detection of sepsis, as shown for abdominal infectious foci [14]. Potential contraindications to the use of x-ray, e.g., in pregnant patients, demand interdisciplinary discussion. Administration of a contrast agent must also be considered cautiously, e.g., in patients with allergy, hyperthyroidism, or kidney failure [15].

Currently, international sepsis guidelines do not make specific recommendations when to choose CT imaging for focus detection [1, 4]. This is due to a lack of CT studies including diagnostic accuracy data in patients with sepsis. Our group previously analyzed CT data of patients with sepsis from different hospital settings in retrospective cohorts [9, 16–18].

This secondary prospective analysis aims to explore the added value of CT for detecting septic foci in patients presenting to the ED with suspected sepsis based on the qSOFA score. We hypothesize that a shorter time-to-CT, i.e., access to earlier CT, may improve patient outcomes in sepsis.

## Methods

### Patient selection

**Fig. 1:**
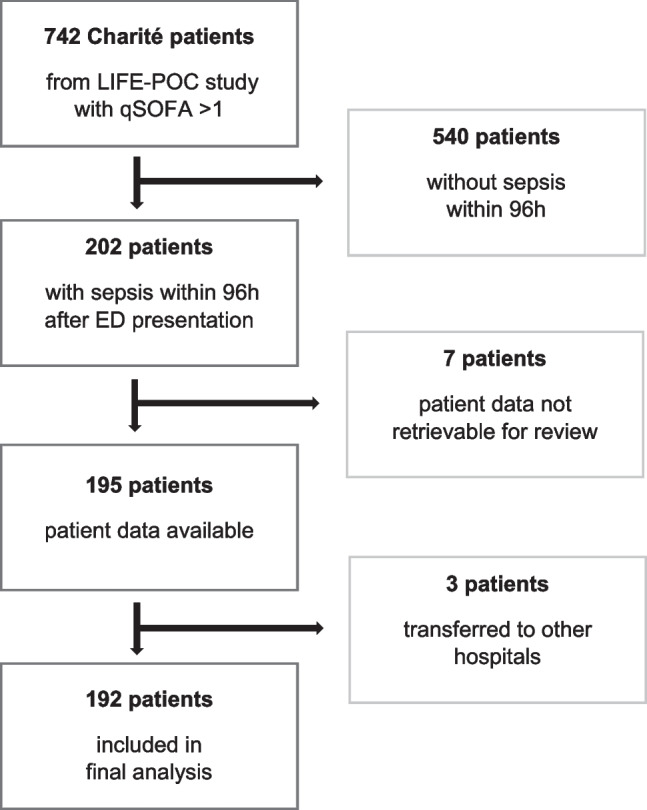
Patient flow chart. A total of 192 patients with radiological data available were included in the present analysis from 742 patients initially screened in the LIFE-POC study. ED, emergency department; qSOFA, quick sequential organ failure assessment

We performed a retrospective secondary analysis of a previously published prospective observational multicenter study, the LIFE-POC study [2]. This study was conducted in three large tertiary hospitals in Germany, the university clinic of Jena, and two sites of Charité University Hospital in Berlin. The original aim of the LIFE-POC study was to identify suitable biomarkers for early sepsis recognition in the ED. Patient recruitment in Berlin took place from 1 January to 23 March 2018. Patients were enrolled 7 days per week in alternating shifts on an 8-h basis. Inclusion criteria were a qSOFA score of at least one, i.e., GCS < 15, blood pressure ≤ 100 mmHg, and respiratory rate ≥ 22/min at the time of ED presentation. Patients were only enrolled once to avoid bias from re-admitted patients. All patients received standard of care in accordance with current guidelines. Exclusion criteria were pregnancy, age < 18 years, and transfer from another hospital. Patients with acute trauma, acute myocardial infarction, suspected acute stroke, or admission for palliative care with a life expectancy of less than 1 month were also excluded. The primary endpoint was the diagnosis of sepsis within 96 h of ED presentation. The diagnosis of sepsis was made by an expert board based on the Sepsis-3 criteria, as mentioned above. In the present study, we included all patients recruited at the two Berlin sites whose radiological data were retrievable at the time of this analysis for review and who developed sepsis within the first 96 h. The secondary analysis of CT reports and patient outcomes was not preplanned. A total of 192 patients were included in the final analysis (Fig. 1). The study was performed in accordance with the ethical guidelines of the Helsinki Declaration (WMA Declaration of Helsinki). Approval was granted by the Institutional Review Board of the University Hospital Jena (4892–08/16). The study was registered with the German registry for clinical trials under DRKS00011188. Written informed consent was obtained from all patients or their legal representatives.

### CT imaging

Patients underwent a CT examination for focus detection, henceforth referred to as focus-CT, based on routine clinical and radiological decision-making. Potential contraindications for CT were evaluated individually on clinical grounds and no recommendations for imaging choice were made by the study protocol. Focus-CT was performed on 64- to 320-row Aquilion scanners ((Canon Medical Systems, USA, formerly Toshiba), i.e., Aquilion ONE Vision (volume-CT)—Aquilion Prime—Aquilion 64—Aquilion Prime) or CT scanners from General Electric (Revolution GSI/EVO 64 Row, Revolution CT (Volume-CT) 256 Row, Light Speed VCT 64 row), and Siemens (Somatom Definition AS 64 Row). Patients with contraindications, e.g., acute kidney injury, allergy, or hyperthyroidism, were given an intravenous contrast agent only after interdisciplinary discussion and with appropriate prophylaxis. Iodine-based intravenous contrast was applied according to institutional standards at least as a mixed portal venous phase as a single series after 60–70 s for combined examinations of chest and abdomen or a split-bolus protocol. Abdominal imaging was performed in at least two phases, e.g., arterial after 40 s, venous after 90 s, if vascular differentials were discussed. In cases that required rule out of pulmonary embolism or bleeding, the contrast phase was automatically triggered by contrast in the pulmonary arteries or aorta, with late venous phase seconds in the latter cases. Neck studies were performed as postinjection protocols with a small field of view and were programed after imaging of the trunk. Cranial CT was generally performed as a native study, and followed by postcontrast studies after a minimum of 180 s in case of suspected cerebral infection. Imaging of the extremities was performed as late venous studies after 90 to 120 s. No oral contrast was applied in this population. Intravenous contrast agents were administered in 71.0% (*n* = 44/62) of the study patients. No adverse events of the contrast application were reported. According to institutional guidelines, CTs for focus search are usually performed with intravenous contrast. However, non-contrast chest CTs may be performed in patients with suspected pneumonia. No other routes of administration were noted in this study. The CT examinations were reviewed by two radiologists independently following institutional routine. Board certification is a requirement for the finalized report. Clinical information may be obtained by all radiologists during reporting. Organ regions scanned by CT were chosen based on clinician’s individual assessment. Singular regions were scanned as opposed to combined regions up to full-body CT scans.

### CT report analysis

Radiological reports and especially CT reports were retrieved from the radiological information system (RIS; GE Healthcare, USA). All reports were studied in detail and categorized using previously published criteria [8]. Three categories were retrieved from the reports: no focus detected, possible focus, or certain focus. The organ region of the focus was noted. The indication for the CT examination was documented as focus search and possibly other indications. Depending on if the patient received a CT scan for focus search or not two groups were built, the CT group and the no-CT group.

In the CT group, the timing of the focus-CT was assessed and confirmed. Information on additional findings was collected. The infectious focus detected by CT was compared with the reference standard defined as the final sepsis focus identified by the treating team of physicians on the basis of the integration of all clinical, imaging, and microbiological findings during hospitalization. The final sepsis focus was retrieved retrospectively from the final medical report of the patient. STARD guidelines were followed. The time-to-CT after sepsis diagnosis (ttCTsd) was noted as well as the time-to-CT after emergency department arrival (ttCTeda). The time of sepsis diagnosis was defined as the time that a SOFA score change of ≥ 2 was clear and the suspicion or certainty of infection was present.

### Patient outcomes

A description of data collection for the overall study population has been reported in detail previously [2]. Briefly, clinical primary data as well as data on the clinical course including patient history, admission date, new infections or organ dysfunction, microbiology, medication, vital signs, laboratory information, and diagnostic data were assessed by the study team and compiled in a study-specific electronic sheet. The electronic or analogous patient records were monitored continuously for four consecutive days or until discharge from the hospital. After 28 days, follow-up telephone interviews were conducted to assess the clinical course and 28-day mortality. Data management and checks for plausibility were performed by the study team of the University of Jena.

### Statistical analysis

Data analysis was performed using SPSS (Version 27, IBM Deutschland GmbH, Ehningen, Germany). Absolute and relative frequencies were calculated. Metric variables were tested for normal distribution using histograms and then summarized as means with standard deviations and compared by using *t* tests in the case of normal distribution. Data which were assessed visually as non-normal were analyzed by using medians and interquartile ranges (ICR) as well as Mann–Whitney *U* test. The chi-square test was performed to compare absolute frequencies and proportions. Diagnostic accuracy was calculated based on cross tables including sensitivity and specificity. With previous data from our group indicating a higher sensitivity than specificity of CT in sepsis, this study aims to confirm the high sensitivity as the primary target. To compare two equally sized groups with short versus long ttCTsd (and ttCTeda), they were separated by the median. Linear regression with the dependent variable ttCTsd was performed with procalcitonin (PCT), Glasgow coma score (GCS), age, and qSOFA score as independent metric variables. Analyses of ttCTsd were similarly performed for ttCTeda as a control (supplementary Table 1). Graphics and tables were built with Microsoft Word and Microsoft Excel (Microsoft Office 16, Microsoft Corporation, Redmond, Washington). A *p* value < 0.05 was considered statistically significant. Due to the exploratory characteristic of this study, no adjustment of *p* values and confidence intervals was conducted. All *p* values and confidence intervals need to be interpreted in a hypothesis-generating manner (Table 1).

**Table 1:** Patient characteristics

	Total	With CT	No-CT	*p *value
Number	192	62	130	
Age median (IQR)	69.0 (57.3–77.0)	64.5 (51.8–73.0)	72.5 (61.8–79.3)	***0.002***
Women in % (*n*)	39.1 (75)	38.7 (24)	39.2 (51)	0.945
qSOFA median (IQR)	1 (1–2)	2 (1–2)	1 (1–2)	0.512
1 in % (*n*)	53.1 (102)	48.4 (30)	55.4 (72)	
2 in % (*n*)	40.1 (77)	46.8 (29)	36.9 (48)	
3 in % (*n*)	6.8 (13)	4.8 (3)	7.7 (10)	
BP systolic < 100 mmHg in % (*n*)	58.3 (112)	64.5 (40)	55.4 (72)	0.230
RR > 22/min in % (*n*)	73.4 (141)	74.2 (46)	73.1 (95)	0.870
GCS < 15 in % (*n*)	21.9 (42)	82.3 (51)	23.8 (31)	0.339
Septic shock				
Day 0 in % (*n*)	9.9 (19)	17.7 (11)	6.2 (8)	***0.012***
Day 1 in % (*n*)	5.2 (10)	9.7 (6)	3.1 (4)	0.070
Day 2 in % (*n*)	5.2 (10)	6.5 (4)	4.6 (6)	0.696
Day 3 in % (*n*)	4.7 (9)	6.5 (4)	3.8 (5)	0.558
Immunosuppression in % (*n*)	21.9 (42)	22.6 (14)	21.5 (28)	0.823
Charlson index* median (IQR)	3 (1–4)	2 (1–4)	3 (2–4)	0.303

Basic patient characteristics are provided as well as sepsis-related clinical data

*BP* blood pressure, *RR* Riva-Rocci, *GCS* Glasgow Coma Scale

## Results

### Patient characteristics

A total of 62 of the 192 patients underwent a CT examination to search for a septic focus within 96 h of ED presentation. The CT group and no-CT group of patients did not differ regarding sex (proportion of women, 38.7% (*n* = 24/62) vs 39.2% (*n* = 51/130), *p* 0.945), qSOFA score (median of 1 (IQR 1–2) vs. 2 (IQR 1–2), *p* = 0.512), and comorbidities (median Charlson index of 3 (1–4) vs. 2 (2–4), *p* = 0.303). However, the median age of patients in the CT group was lower than in the no-CT group (median of 64.5 years (IQR, 51.8–73.0) vs. 72.5 years (IQR 61.8–79.3), *p* = 0.002). Furthermore, septic shock occurred more often in the CT group in comparison to the no-CT group (17.7% (*n* = 11/62) vs. 6.2 (*n* = 8/130), *p* = 0.012). The most common final septic focus was pulmonary (51.0%; *n* = 98/192), followed by abdominal foci in 10.9% (*n* = 21/192), and other foci (Table 2).

**Table 2:** Infectious foci

Final septic focus	All patients (*n* = 192)	Patients with CT (*n* = 62)	Patients without CT (*n* = 130)
Pulmonary focus in % (*n*)	52.0% (100)	43.5% (27)	56.2% (73)
Abdominal focus in % (*n*)	13.0% (25)	21.0% (17)	6.2% (8)
Pancreas in % (*n*)	2.1% (4)	6.5% (4)	0% (0)
Gastrointestinal in % (*n*)	2.6% (5)	4.8% (3)	1.5% (2)
Hepatobiliary in % (*n*)	2.1% (4)	3.2% (2)	1.5% (2)
Peritonitis in % (*n*)	0.5% (1)	1.6% (1)	0% (0)
Unknown focus in % (*n*)	7.8% (15)	8.0% (5)	7.7% (10)
Urogenital focus in % (*n*)	15.6% (30)	4.8% (3)	20.8% (27)
Other diagnoses in % (*n*)	6.8% (13)	11.3% (7)	4.6% (6)
Soft tissue in % (*n*)	4.7% (9)	8.0% (5)	3.0% (4)
Bacteremia in % (*n*)	3.1% (6)	6.5% (4)	1.5% (2)

The table presents results for the final sepsis focus and infectious foci detected by CT

In the CT group, the majority of the patients 43.5% (*n* = 27/62) had a pulmonary focus. Of these patients, 56% (*n* = 15/17) presented with atypical pneumonia, 33% (*n* = 9/27) with typical pneumonia, and for 11% (*n* = 3/27) patients it was not specified. Among the most common abdominal sources of sepsis were retroperitoneal abscesses (*n* = 6/17), colitis/gastroenteritis (*n* = 5/17), and pancreatitis (*n* = 4/17). Less common were cholecystitis, diverticulitis, spontaneous bacterial peritonitis, etc. In four patients, abdominal emergencies, specifically perforations, were reported. No postsurgical patients were identified in the no-CT group.

In the CT group (*n* = 62/192), 96.8% of the patients received a microbiological test to identify the responsible agent. In 20%, no agent was identified. A detailed description of the etiological agents can be found in the supplementary Table 2.

### CT report analysis

**Fig. 2:**
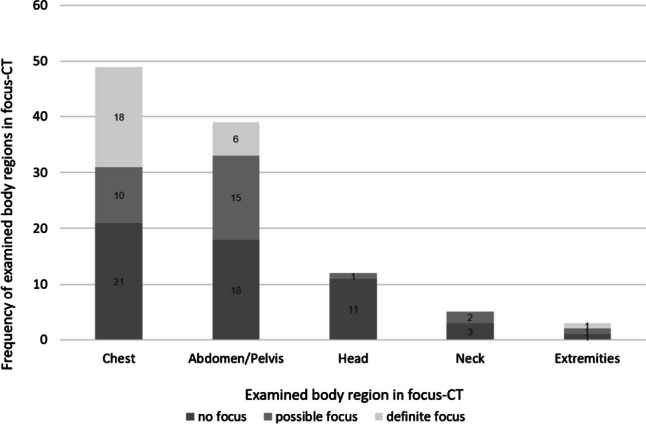
Diagnostic certainty and body regions examined by focus-CT in the CT group of patients (*n* = 62/192). Diagnostic certainty indicates whether there was no focus detected, a possible or a definite infectious focus based on the radiological report

The median time-to-CT after sepsis diagnosis (ttCTsd) was 3.83 h (IQR 1.50–29.97 h), whereas the median time-to-CT after emergency department arrival (ttCTeda) was 7.44 h (IQR 3.68–30.77 h). With regard to organ regions covered by the CT scan, approximately half of the patients (*n* = 32) received a CT scan of more than one organ region. Specifically, in 33.9% of the patients (*n* = 21), more than two organ regions were scanned, in 12.9% (*n* = 8), more than 3, and in 4.8% (*n* = 3), four organ regions were scanned. The most commonly examined region was the chest (95.2%; *n* = 49/62), followed by the abdomen/pelvis (62.9%; *n* = 39/62), and the head (19.4%; *n* = 12/62). The extremities were the organ region least commonly examined at 4.8% (*n* = 3/62) (Fig. 2). The percentage of positive findings on CT, i.e., graded as possible or certain foci, per organ region was 57.1% (*n* = 28/49) for the chest and 53.9% (*n* = 21/39) for abdomen/pelvis. An infectious focus was least commonly found in the head (8.3%, *n* = 1/11). In 58.1% (*n* = 36/62) of focus-CTs, secondary findings were documented. Most commonly, other findings (55.6%; *n* = 20/36), tumor (11.1%; *n* = 4/36), and trauma (8.3%; *n* = 3/36) were noted. Only one patient had a fracture as an incidental finding. In 25.0% (*n* = 9/36), several secondary findings were documented. In 41.9% (*n* = 26/62), no secondary findings were noted.

Comparison of infectious foci between the CT group versus the no-CT group revealed that performing a focus-CT was significantly associated with the final septic focus being pulmonary or intraabdominal, with *p* < 0.0001 (Table 2). Patients with no-focus-CT showed a significant association with a urogenital focus or unclear death with *p* < 0.0001.

### Diagnostic accuracy

**Fig. 3:**
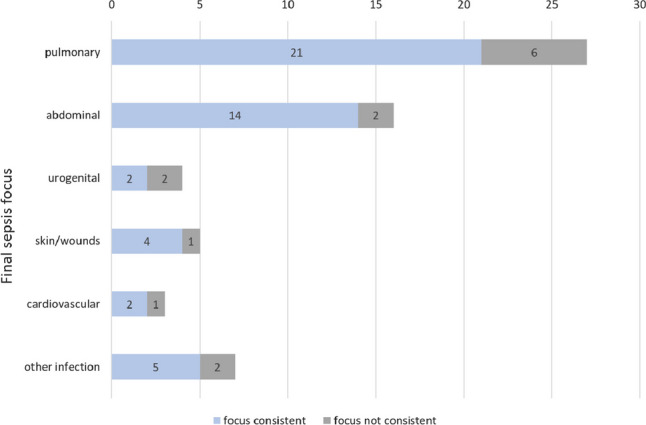
Infectious focus consistency between focus-CT and final septic focus of the CT group of patients (*n* = 62/192)

Among patients who received a CT scan, the most common infectious foci that led to sepsis were pulmonary (43.3%; *n* = 27), abdominal (25.8%; *n* = 16), and urogenital (6.4%; *n* = 4). The consistency of the final sepsis focus with the focus detected by CT was 77.8% (*n* = 21/27) when the focus was pulmonary and 87.5% (*n* = 14/16) when the focus was abdominal. In patients with urogenital sepsis, the focus was confirmed in 50% (*n* = 2/4) of the cases by CT (Fig. 3; Table 2). For focus detection, a sensitivity of 81.1% (95% confidence interval (CI), 68.0–90.6%) was calculated, with a specificity of 55.6% (95% CI 21.2–86.3%).

### Patient outcomes

Surgical source control, e.g., debridement of infected tissues, was performed in 10.8% (*n* = 21/192) of the patients within 96 h after ED admission. Patients with CT underwent surgical source control more often than patients with no-focus-CT (20.9% vs 6.1%; *p* = 0.02). Thirteen percent (*n* = 25/192) of all patients died within 28 days after study enrollment. The length of hospital stay was found to be longer in the focus-CT group (median 15 d, IQR 10 d–24 d vs median 9 d, IQR 7 d–14 d 06 h; *p* = 0.001). Mortality did not differ significantly between the focus-CT (12.9%, *n* = 8) and no-focus-CT group (13.1, *n* = 17; *p* = 0.973) (Table 3) or between patients with a short ttCTsd versus long ttCDsd (16.1%, *n* = 5/31 vs 9.7, *n* = 3/31; *p* = 0.449) (Table 4). Median survival time in patients with a short ttCTsd (17 days; IQR 4 d 12 h–23 d 00 h) and long ttCTsd (8 days; IQR 1 d 00 h–11 d 00 h) did not differ with *p* = 0.651. There were no differences between short and long ttCTsd regarding length of hospital stay (median 16 days, IQR 9 d 12 h–23 d 18 h vs median 13 days, IQR 10 d 00 h–24 d 00 h; *p* = 0.863) or duration of intensive care (median 3 d 12 h, IQR 2 d 6 h–7 d 18 h vs median 5 d, IQR 2 d–11 d; *p* = 0.800). Linear regression analysis of relevant covariates revealed that higher PCT and lower GCS were associated with lower ttCTsd, with (beta =  − 839.80, *p* = 0.551 and beta = 12,328.93, *p* = 0.261), respectively. Higher age and higher qSOFA score were associated with higher ttCTsd (beta = 2334.99, *p* = 0.218 and beta = 57,919.86, *p* = 0.240), respectively (supplementary Table 2).

**Table 3:** Morbidity and mortality of patients with sepsis in the ED for CT group and no-CT group

	CT	No-CT	*p* value
Hospital length of stay			
Mean	15 d 21 h	11 d 03 h	**0.001**
SD	7 d 21 h	7 d 16 h	
ICU length of stay			
Median	4 d 00 h	4 d 00 h	0.914
IQR	2 d 00 h–10 d 00 h	2 d 00 h–9 d 00 h	
Death within 28 days			
in % (portion/total)	12.9 (*n* = 8/62)	13.1 (*n* = 17/130)	0.973
Time to death within 28 days			
Median	12 d 12 h	4 d 00 h	0.150
IQR	2 d 18 h–22 d 18 h	1d 12 h–14 d 00 h	

*IQR* interquartile range, *SD* standard deviation

**Table 4:** Morbidity and mortality of patients with sepsis in the ED according to time-to-CT after sepsis diagnosis (ttCTsd)

	ttCTsd		
	< 3.83 h	> 3.83 h	*p* value
Hospital length of stay			
Mean	16 d 04 h	15 d 15 h	0.863
SD	7 d 22 h	8 d 02 h	
ICU length of stay			
Median	3 d 12 h	5 d 00 h	0.800
IQR	2 d 6 h–7 d 18 h	2 d 00 h–11 d 00 h	
Death within 28 days			
in % (portion/total)	16.1 (*n* = 5/31)	9.7 (*n* = 3/31)	0.449
Time to death within 28 days			
Median	17 d 00 h	8 d 00 h	0.651
IQR	4 d 12 h–23 d 00 h	1 d 00 h–11 d 00 h	

*IQR* interquartile range, *SD* standard deviation

## Discussion

### Summary of findings

In the majority of septic patients included in our secondary analysis, the final septic focus was recognized on CT. We found a high sensitivity and a lower specificity for focus-CT. Regarding the effect of time-to-CT on mortality, length of hospital, and ICU stays in septic patients, no differences were observed for any endpoints investigated between the short ttCDsd group and the long ttCDsd group.

### Comparison with published findings

These data confirm previous findings by our group pointing towards the value of CT in its high sensitivity for sepsis focus identification, which was even higher in the present analysis than previously reported [8, 16, 17]. The current data again show a low specificity, also consistent with prior studies. This means that CT scans should not be performed to exclude septic foci, but to identify them on the basis of clinical suspicion. The latter may not apply in sepsis cases with urogenital focus as confirmed in our study. Although urogenital foci are common infectious sources in sepsis, CT imaging may not have a major role in detecting them [9]. Regarding pulmonary foci of sepsis, our data support those of previous studies that showed the added value of CT chest to detect pneumonia in the emergency department [10]. We previously analyzed the findings of repeat CTs, which may reveal a focus in initially negative focus-CT. Even though other authors did not provide accuracy data for CT in sepsis, there are some publications on focus search in sepsis [13, 19]. Both Schleder et al and Just et al reported marginally lower rates of positive findings on focus-CT as compared with our data. The added value of this study is the analysis of CT in prospectively recruited patients with sepsis with excellent clinical descriptions as compared with previously published retrospective cohorts by this and other groups.

To our knowledge, we present here the first study investigating time-to-CT (ttCTsd) in sepsis with the aim to quantify a benefit in patient outcomes such as mortality and morbidity. No advantage was shown for the early-CT group versus the late-CT group or for the CT group versus the no-CT group. These findings may partly be attributed to differences in clinical condition at the time when a CT was considered, as the decision to perform a CT and its priority were discussed between the treating physician and radiologist. Thus, patients with more severe disease and associated morbidity might have undergone a CT earlier as a consequence. This procedure might have undermined the advantage of an earlier CT, i.e., the clinical prioritization might have been a possible confounder in our study population. Prospective randomized studies with proper control for all confounding factors are needed to determine the benefits of an early CT. Even though a recent survey by our group points to the high relevance of CT timing in sepsis for different disciplines [20], this topic remains understudied.

### Limitations

First, this analysis is limited by its small sample size, imbalances between subgroups, and differences in the baseline characteristics due to its observational study design. Therefore, part of the statistical analysis may be underpowered and conclusions require confirmation by larger studies. Second, as the reference standard final sepsis focus may be influenced by imaging, a bias may be presumed. However, this reference standard applies to both focus-positive and focus-negative CTs analyzed. The interpretation of the accuracy data should be considered limited. Still, the study population is very well characterized with detailed clinical descriptions. The statistical analysis should be seen as exploratory rather than confirmatory. Furthermore, for ethical reasons, not all patients with dementia or cognitive deficits were included. Consequently, fewer patients with a qSOFA score of three were included, as GCS is part of the qSOFA assessment. Preferably, a randomized trial would avoid bias due to non-inclusion of certain patient groups. However, this study allowed a high level of surveillance that other study setups might not provide. Finally, this study does not allow conclusions on which patients with sepsis should receive CT or not. The decision-making process for the individual imaging choice in each patient is not documented in this study and the study protocol did not influence imaging decisions. Even though criteria for evidence-based imaging choice in sepsis are still missing, this is beyond the scope of this manuscript.

## Conclusion

Our results show a high sensitivity of CT in focus detection in ED patients with sepsis, showing its added value in guiding treatment decisions. The low specificity suggests that a negative CT requires further ancillary diagnostic tests such as microbiology for focus detection. The timing of CT did not affect morbidity or mortality outcomes in our secondary analysis. More guidance for diagnostic imaging choices in sepsis is required which should rely on prospectively randomized generated outcome research.

## Supplementary Information

Below is the link to the electronic supplementary material.

Supplementary file1 (PDF 283 KB)

## Declarations

### Guarantor

The scientific guarantor of this publication is Myrto Bolanaki.

### Statistics and biometry

One of the authors, K.R., has significant statistical expertise.

### Study subjects or cohorts overlap

Some study subjects or cohorts have been previously reported in our published article “Diagnostic Performance of Procalcitonin for the Early Identification of Sepsis in Patients with Elevated qSOFA Score at Emergency Admission.” Bolanaki M, Möckel M, Winning J, Bauer M, Reinhart K, Stacke A, Hajdu P, Slagman A. J Clin Med. 2021 Aug 28;10(17):3869. 10.3390/jcm10173869. PMID: 34501324; PMCID: PMC8432218.

### Methodology

- prospective observational multicenter study
