# Supplementary material for: Computed tomography in patients with sepsis presenting to the emergency department: exploring its role in light of patient outcomes
Source: Eur Radiol. 2024 Apr 9;34(10):6466–74. doi: 10.1007/s00330-024-10701-y (PMC11399293; doi:10.1007/s00330-024-10701-y)
Supplement: Supplementary file 1 — Supplementary file1 (PDF 283 KB) [file 330_2024_10701_MOESM1_ESM.pdf]

Computed tomography in patients with sepsis presenting to the emergency department: exploring its role in light of patient outcomes

ELECTRONIC SUPPLEMENTARY MATERIAL

Supplementary Table 1: Morbidity and mortality of patients with sepsis in the ED by time-to-CT after emergency department arrival (ttCTeda).

IQR – interquartile range; SD – standard deviation.

	**ttCTeda**		
	**<7.44h**	**>7.44h**	***p*-Value**
**Hospital length of stay**			
Mean	14d 22h	16d 16h	0.555
SD	8d 03h	7d 19h	
**ICU length of stay**			
Median	4d 00h	3d 00h	0.636
IQR	2d 00h - 8d 00h	2d 00h - 15d 00h	
**Death within 28 days**			
in % (portion/total)	12.9 (n=4/31)	12.9 (n=4/31)	1.000
**Time to death within 28 days**			
Median	19d 12h	8d 00h	0.465
IQR	5d 00h - 23d 12h	2d 18h - 19d 06h	

Supplementary Table 2: Multiple linear regression model analyzing the impact of clinical sepsis-related factors on ttCTsd.

Coefficients

	unstandardized coefficients		standardized coefficients		
**model**	**B**	**Std. Error**	**Beta**	**T**	**p**
constant	-321350.78	228947.65		-1.40	*0.166*
age	2334.99	1875.63	0.16	1.24	*0.218*
procalcitonin	-839.80	1398.83	-0.81	-0.60	*0.551*
GCS	12328.93	10869.97	0.15	1.13	*0.261*
qSOFA score	57919.85	48758.89	0.16	1.18	*0.240*

Abbreviations: GCS - Glasgow coma scale; qSOFA - quick sequential organ failure assessment; ttCTsd - time-to-CT after sepsis diagnosis

Supplementary Table 3: Frequency of bacterial etiologies among patients in CT group that received a microbiological test.

**Etiological agents**	**Frequency**	**Percentage**
**Gram positive**	11	17.7
**Gram negative**	15	24.2
**Fungal**	6	9.7
**Parasites**	1	1.6
**Multiple agents**	7	11.3
**Negative**	20	33.3
**Total**	60	100

Supplementary table 4: Detailed oncological comorbidities of the study population

	**Total**	**With CT**	**No-CT**
**Number**	192	62	130
**Solid tumor** in % (n)	12.0 (23)	12.9 (8)	11.5 (15)
**Metastatic Tumor** in % (n)	12.5 (24)	14.5 (9)	11.5 (15)
**Leukemia** in % (n)	2.6 (5)	3.2 (2)	2.3 (3)
**Lymphoma** in % (n)	6.6 (12)	4.8 (3)	6.9 (9)
